# Protocol to analyze transmigration of human cytotoxic T cells under physiological flow conditions *in vitro*

**DOI:** 10.1016/j.xpro.2022.101509

**Published:** 2022-06-30

**Authors:** Rouven Schoppmeyer, Jaap D. van Buul

**Affiliations:** 1 Molecular Cell Biology Lab, Department of Molecular Hematology, Sanquin Research, Amsterdam, the Netherlands; 2 Landsteiner Laboratory, Amsterdam UMC, University of Amsterdam, Amsterdam, the Netherlands; 3 Leeuwenhoek Centre for Advanced Microscopy (LCAM), Section Molecular Cytology at Swammerdam Institute for Life Sciences (SILS) at University of Amsterdam, Amsterdam, the Netherlands

**Keywords:** Cell Biology, Immunology, Microscopy

## Abstract

This protocol presents an assay for transmigration analysis of human cytotoxic T cells (CTL) under physiological flow *in vitro*. We describe detailed analysis steps of human CTL behavior, from adhesion to diapedesis, using live cell imaging which cannot be achieved by *in vivo* imaging. The flow system is made of 2D plastic surfaces covered by an endothelial monolayer limiting the system but allows for quantitative analysis of CTL behavior with high modifiability.

For complete details on the use and execution of this protocol, please refer to Schoppmeyer et al. (2022).

## Highlights

• *In vitro* live cell imaging of transmigrating human T cells
• Preparation of inflamed endothelial monolayers and flow system setup
• Quantitative data on CTL adhesion, transmigration, and route of transmigration

## Abstract (editor highlights)

Publisher’s note: Undertaking any experimental protocol requires adherence to local institutional guidelines for laboratory safety and ethics.

## Graphical abstract

**Figure:**
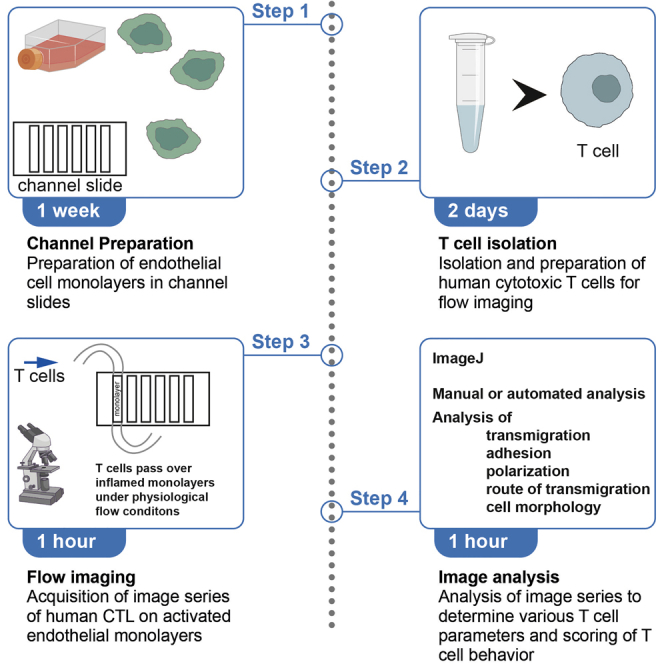

## Before you begin

### Institutional permissions

For isolation of human cytotoxic T cells (CTL) fresh blood in heparin collection tubes or buffy coats from transfusion bags are required. We receive our blood donations from healthy voluntary donors. Local regulations and requirements may apply for the isolation and use of human blood and blood-derived cells in research applications.

### Culture of endothelial cells

**Timing: 1 week**

1. Thaw of frozen endothelial stocks.
  ***Note:*** We usually thaw endothelial cells on Tuesdays for them to be seeded for experiments on the following week.
  a. Coat plastic culture dishes (T150 flask or 10 cm dishes) using 5 mL fibronectin solution (Sanquin Reagents) for 1 h at 37°C, 5% CO_2_ in an incubator, aspirate.
    i. Wash using 10 mL PBS, aspirate.
  b. Thaw endothelial cells by taking them from liquid nitrogen and circle the tube slowly inside a 37°C water bath until the frozen part detaches and a small clump of ice remains floating in the thawed medium.
  c. Wipe the tube thoroughly with 70% ethanol and pour the inside of the tube into 10 mL full EGM-2.
    i. Centrifuge at 100 g for 10 min and remove supernatant.
    ii. Add 10 mL (10 cm dish) or 15 mL (T150 flask) of full EGM-2 to the cells.
2. Seed cells.
  a. Transfer cell suspension into the fibronectin-coated dish or flask (day 0).
  b. Place in an incubator at 37°C 5% CO_2_.
  c. Refresh medium the day after seeding (day 1), leave in culture for 2 more days (until day 3).
  d. On day 3 after seeding, split cells by trypsinization.
    i. Coat the required number of flasks or dishes using fibronectin solution.
      ***Note:*** The given protocol and numbers will yield about 1.5 × 10^6^ HUVEC cells per 10 cm dish or T150 flask after 3 days of culture (**proliferation rate can differ between HUVEC pools!**).
    ii. Wash dish or flask 2 × using 15 mL PBS, aspirate.
    iii. Add 1 mL trypsin∗EDTA solution, incubate for 3–5 min.
    iv. Add 1 mL trypsin neutralizing solution.
    v. Harvest and centrifuge (100 g, 10 min) in a 15 mL tube.
    vi. Resuspend to 1 × 10^6^ cells per mL.
    vii. Seed 250.000 cells per 10 cm dish or T150 flask in 10 mL or 15 mL EGM-2.
  e. Culture for 3 more days and split cells again to seed to final culture or experimental dishes.

### Isolation of human CTL and recovery culture

**Timing: 1 day before the experiment**

3. Isolate human PBMC from fresh human heparinized blood.
  **CRITICAL:** Perform all steps with cold buffers and on ice (except centrifugation: use RT).
  a. Dilute fresh heparinized blood 1:1 with PBS +2 mM EDTA (PBMC buffer, cold).
  b. Prepare CTL isolation buffer: PBS + 2 mM EDTA + 0.5% FCS (cold).
  c. Layer 15 mL of Ficoll Paque PLUS into 50 mL conical bottom tubes.
  d. Carefully layer 35 mL of diluted blood on top of the Ficoll layer without mixing the phases.
    i. Tilt the tube and slowly empty the pipette onto the tube wall so that Ficoll layer and blood layer do not mix.
  e. Centrifuge at 400 g for 40 min at RT without break, acceleration can be left on high.
    i. If you are using separation tubes like Leukosep or SepMate please stick to the recommendations of the supplier as centrifugation times, break settings and speed will differ.
  f. Remove the upper (yellowish) layer of plasma until 5–10 mL are left on top the white PBMC ring.
  g. Remove the PBMC ring with a pipette and transfer into a fresh tube to be filled to 50 mL using PBMC buffer.
    i. Centrifuge at 350 g for 10 min and remove supernatant.
  h. Resuspend pellet in 50 mL PBMC buffer and centrifuge at 200 g for 10 min.
    i. Take a sample to count the cells at this point.
    ii. Repeat the washing step once.
  i. After the final centrifugation remove the supernatant and resuspend pellet in isolation buffer according to the instructions of the Miltenyi CD8^+^ isolation kit.
4. Isolate human CD8^+^ T cells from PBMC.
  a. Follow the instructions of the Miltenyi CD8^+^ isolation kit.
    i. CTL isolation buffer: PBS + 2 mM EDTA + 0.5% FCS (cold).
    ii. Use LS columns with the mentioned kit.
5. Ensure the following: Recovery conditions for human CD8^+^ T cells.
  a. Recovery medium for CTL: RPMI1640 + 10% FCS + 1% Pen/Strep + 10 mM HEPES.
  b. Recovery density of CTL: 2 × 10^6^ mL^-1^.
  c. Recovery time of CTL: overnight at 37°C and 5% CO_2_.

**CRITICAL:** CTL yield from fresh PBMC can range from 5 to 20% of the total PBMC amount. Calculate input PBMC by assuming the lowest yield (5%) to determine how many PBMC you need for isolation to yield at least the amount of cells required (1 × 10^6^ CTL per flow channel). Do not use less than 10 × 10^6^ PBMC as input for CTL isolation. Most critical are the recovery culture conditions, more specifically the pH stability which can be reduced drastically for older RPMI medium. The addition of HEPES (10 mM) to RPMI avoids this (troubleshooting 5). Expect some degree of variation in transendothelial migration parameters between differing imaging frames, as effector and effector memory frequency can vary between donors (Schoppmeyer et al., 2022). If effector and EM numbers are extremely low adhesion numbers will drop whilst other parameters are not reduced or inhibited.

### Seeding and stimulating HUVEC in the flow channel system

**Timing: 1 h before seeding endothelial cells into flow channels**

6. Coat flow channels with fibronectin.
  ***Note:*** The handling of μ-channels requires some practice, but the manufacturer provides excellent manuals (https://ibidi.com/channel-slides/57--slide-vi-04.html), we will cover the most important points to note.
  a. Coat μ-channel VI 0.4 using fibronectin solution (other channel sizes are available).
    i. For easy transport place the μ-channel into a 10 cm petri dish.
    ii. Use a 100 μL or 200 μL pipette for loading cells or applying coating solution.
      ***Optional:*** To avoid air bubbles from forming in the channel, degas your solutions e.g., in a vacuum chamber. Equilibrating media and buffers prior to their use in the channel system reduces air inclusions already drastically.
    iii. Load 30 μL (per channel) of coating solution by pressing the pipette tip into the channel entry and release continuously in one fast motion (Figures 2E–2G).

**Figure 1:**
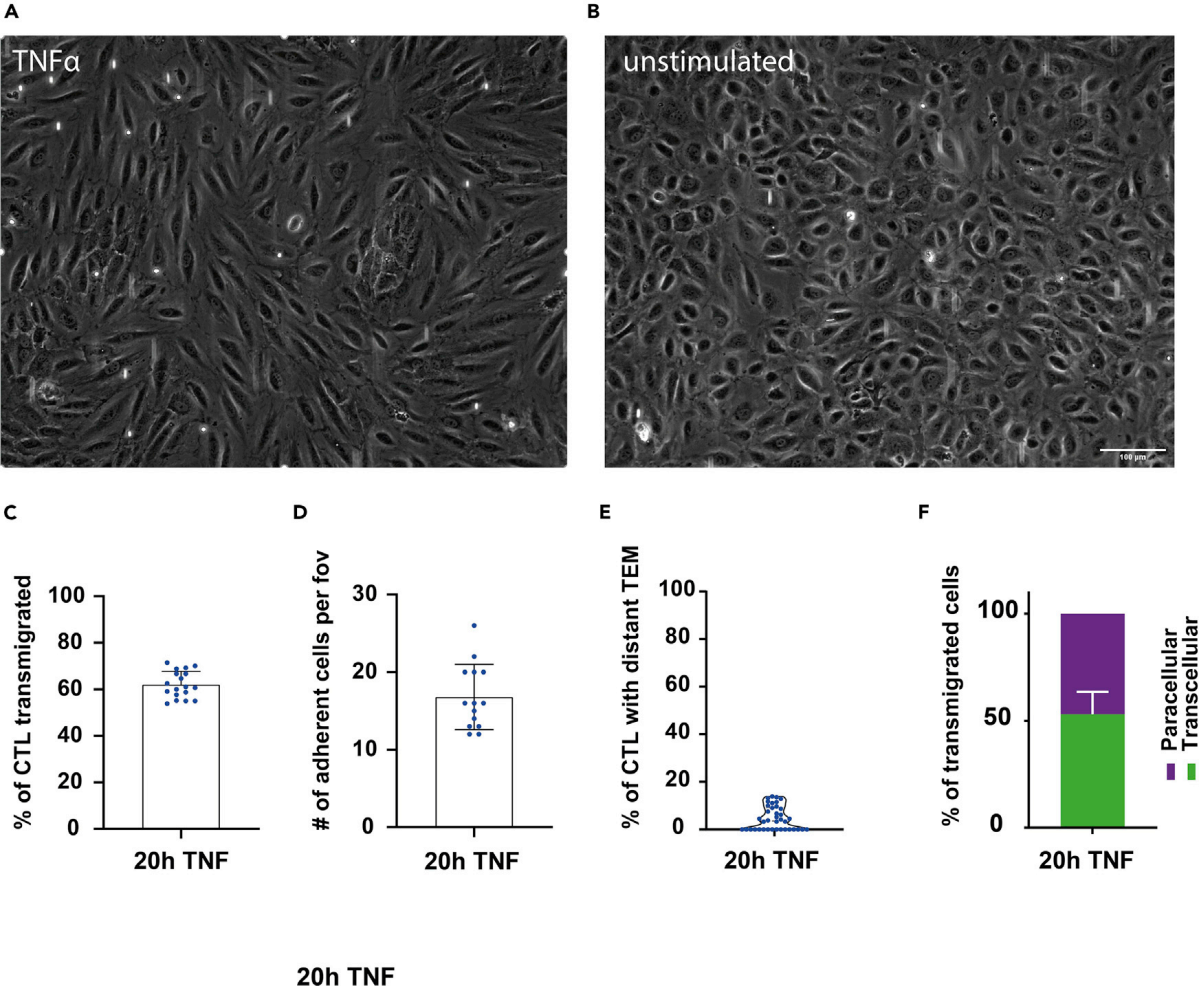
Example transmigration analysis of human CTL on 20 h TNFα-activated HUVEC (A and B) A HUVEC monolayer in a μ-channel VI 0.4 is shown with and without inflammatory stimulation for 20 h. White spots in a are bypassing CTL. Images were taken after cell injection but before cell adhesion occurred. (C–F) example results of a control experiment prepared as described in this protocol. Note the different morphology of the HUVEC cells after inflammatory stimulation. For A see Methods video S1 and for B see Methods video S2 (related to: Image analysis and quantification of transmigration parameters). Error bars are standard deviation calculated by graphpad prism 9. Scale bar represents 100 µm (A and B at same magnification).

**Figure 2:**
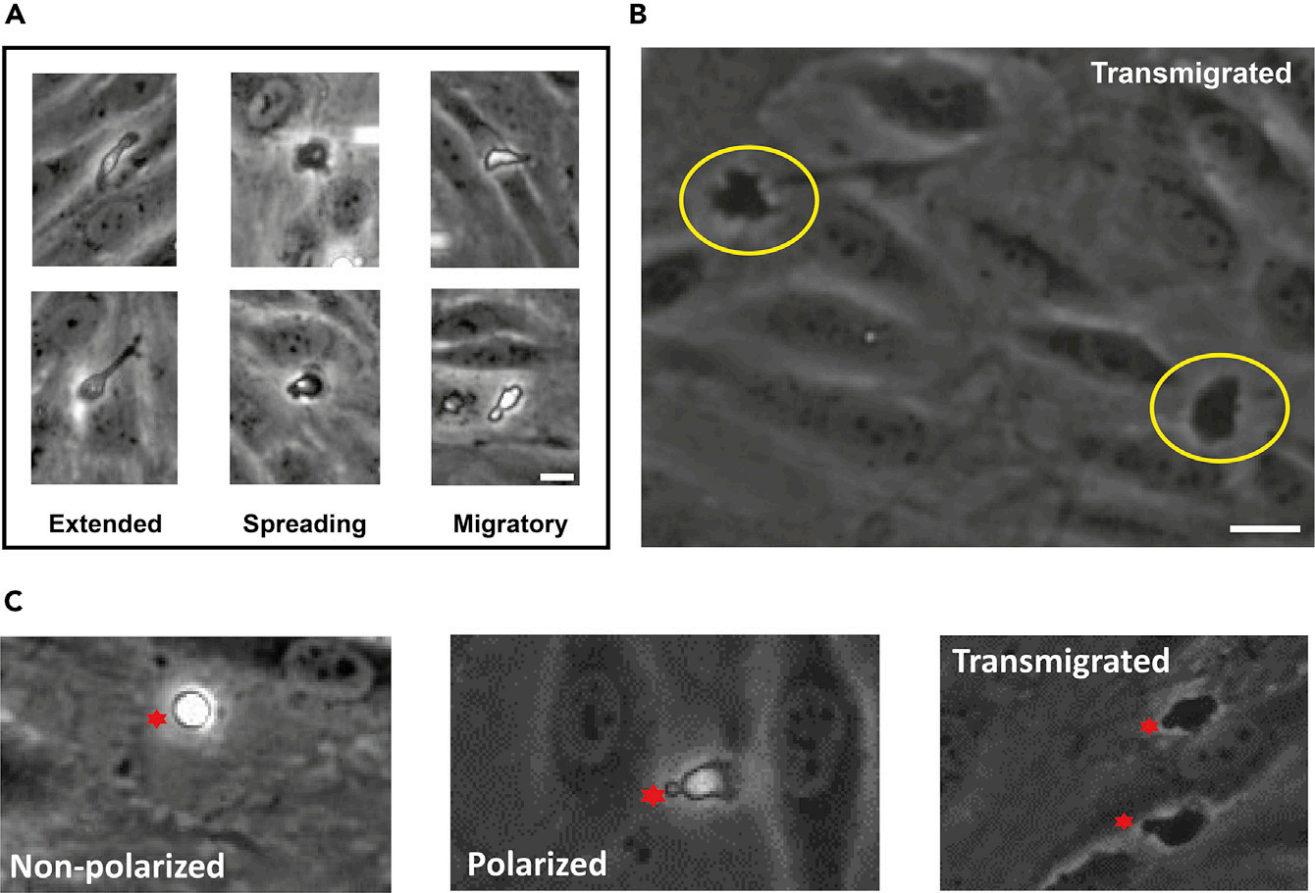
Morphologies of CTL contacting inflamed HUVEC monolayers (A) The morphologies usually observed for CTL directly after adhesion to inflamed HUVEC monolayers from flow are shown. **Extended** is a rare case where CTL detachment seems affected. If this occurs in high frequency in controls troubleshoot CTL and HUVEC culture conditions and inflammation efficiency. The **spreading** phenotype can often be observed for transcellular transmigrating CTL. In those cases the spreading is quickly followed by a depolarization (returns to rounded cell morphology, examples in Methods video S3). The **migratory** phenotype results in apical migration of adhering CTL and represents the classical CTL morphology when migrating in a tissue matrix or 2D systems (Schoppmeyer et al., 2017; Zhou et al., 2018). This morphology precedes paracellular events but CTL also adapt this morphology and migrate apically without transmigrating. Scale bar, 10 µm. (B) When transmigrated CTL change contrast to a homogenous dark appearance. Examples are indicated by circles. Note that in still images transmigrated CTL are difficult to identify but very obvious in image series. Thus we always recommend recording a series over 20 min at as low intervals as 1 up to 10 s (see Methods videos S4 and S5). Scale bar, 10 µm. (C) shows the most common parameters important for general analysis: non-polarized (chemotactic stimulation), apically migrating (polarized) and transmigrated CTL. Red asterisks mark CTL (related to: Image analysis and quantification of transmigration parameters).
      Methods video S1. TNF control example: 20 h TNFα treated HUVEC monolayer flown with 1 × 10^6^ recovered CTL, related to Imaging transmigration under flow
      Methods video S2. Unstimulated control example: Unstimulated HUVEC monolayer flown with 1 × 10^6^ recovered CTL, related to Imaging transmigration under flow
      Methods video S3. Paracellular example: Example of a human CTL transmigrating via the paracellular route, showing polarization and apical migration on the HUVEC cell of adhesion and performs paracellular migration on a near EC:EC junction, related to Image analysis and quantification of transmigration parameters Before transmigrating the CTL takes a migratory morphology and maintains this before and during paracellular diapedesis.
      Methods video S4. Transcellular example: Example of a human CTL transmigrating via the transcellular route identified by lack of polarization and apical migration after contact and undirected spreading post TEM, related to Image analysis and quantification of transmigration parameters These parameters where identified to be specific for transcellular events using high resolution fluorescence imaging (Schoppmeyer et al., 2022).
      Methods video S5. Opening connected flow tubing: Opening the flow system, related to Figure 3 Open the clamps on both sides of the tubing only when the system is connected and sealed (all Luer locks connected to syringe, channel and that the intake tubing is at the bottom of the liquid container).
      Methods video S6. Pump setup: Flow system fully set up, related to Figure 3 and Preparation of the flow system Liquid container and pump are connected via the channel.
      Methods video S7. Closing the clamps: Clamping the tubing, related to Figure 3 and Preparation of the flow system Clamp tubing when changing channels or emptying the syringe when full. Fully push the clamp to the end. Never clamp during an experiment. Always check twice before opening the clamps if all connections are sealed.
      Methods video S8. Flush the pump system: Activating the flow system, related to Figure 3 and Preparation of the flow system After connecting the primed tubing open the clamps and activate the pump. Observe for trapped air bubbles flowing through the system to identify potential leaks. Flow endothelial cells for at least 10 min before injecting CTL.
      Methods video S9. Flat untransmigrated CTL: Example of a “false positive” TEM event, related to Image analysis and quantification of transmigration parameters Circle marks area of CTL entry into the frame. The CTL shows homogenous dark contrast and becomes bright for a brief period later in the image series. This cell is not transmigrated and each events should be observed individually over the whole course of the experimental run to ensure to count the proper events.
      Methods video S10. Morphology examples of CTL under flow: Morphology examples rounded, spreading, extended and polarized taken by cells in succession, related to Image analysis and quantification of transmigration parameters Cells also flatten showing inhomogeneous reduction of contrast which is not to be mistaken for transmigration. Note that none of the cells in this video have actually transmigrated! For this experiments endothelial cells were pretreated with an anti-ICAM-1 blocking antibody.
      Methods video S11. Common CTL morphologies, static: Morphology examples of rounded, polarized and transmigrated CTL under static conditions to emphasize the general morphologies, related to Image analysis and quantification of transmigration parameters Samples are prepared identical as for flow assays yet CTL are added to the channel by medium exchange with CTL suspension without application of flow. Note that, due to thermic drift caused, which is common for microscopic incubation chambers, rounded and usually immotile cells show drifting movement. For the same reasons CTL gather towards the upper left corner (center of slide). Transmigrated cells show high motility after transmigration but can only move laterally. The image series was recorded after transmigration events had mostly completed (10 min after addition of CTL). Naïve CTL make up the majority of total CTL isolates and naïve cells show no adhesion to TNFα activated HUVEC. Due to this static experiments (as shown in this movie) show lower transmigration efficiencies compared to flow based assays.
    iv. Place channel for 1 h at 37°C, 5% CO_2_ in an incubator.
    v. Aspirate using a cell culture aspirator by completely removing all liquid from the channel.
      ***Note:*** Apply aspirator to channel entry only when removing coating solution.
    vi. For a medium change of live cells aspirate ONLY at the opposite side of the channel entry in the reservoir, to not aspirate the medium in the channel when emptying the reservoir.
      **CRITICAL:** Do not remove the medium from the channel containing live cells (it will kill the cells immediately). We recommend to avoid this.

7. Seed endothelial cells.
  a. Trypsinize endothelial cells as described above 6 days after thawing.
    ***Note:*** Endothelial cells should have reached about 70% confluence at time of trypsinization.
  b. Set cell density in EGM-2 to 1 × 10^6^ mL^-1^.
  c. Seed 30 μL of this cell suspension into the coated μ-channel VI 0.4.
    ***Note:*** Apply cell suspension in one fluid eject motion from the pipette to avoid air inclusions as cells do not grow at sites of air inclusion disrupting the monolayer.
  d. Incubate for 20–30 min in an incubator at 37°C and 5% CO_2_.
  e. Visually check cell attachment using a light microscope (cells spread out on the surface) and add 120 μL EGM-2 to each channel through one of the inlet ports.
  f. Gently rock the channel twice to ensure medium levels are equal in both inlets and ensure that the medium does not stick to the lid.
    ***Note:*** This can happen if the medium does not level out due to a blocked opposite inlet, slow rocking (before adding the lid) will resolve this.
  g. Culture for 2 days and change medium every day.
    i. Aspirate medium by using a cell culture aspirator.
      ***Note:*** When aspirating (using a 200 μL tip attached to the tip of a glass Pasteur pipette or normal pipette) never touch the channel inlet itself.
      **CRITICAL:** After cells are seeded in the channel and you want to e.g., change medium DO NOT remove the medium within the channel itself.
      **CRITICAL:** Only aspirate the reservoir (contains ∼ 60 μL) using a 200 μL pipette tip. Touching the channel entry with an active aspirator will empty it immediately and kill all the cells.
      ***Alternatives:*** you can use a pipette to manually empty the reservoir but also on the opposite side of the channel entry within the reservoir.
    ii. Add 120 μL EGM-2 into one of the reservoirs.
8. Induce of inflammation in endothelial monolayers.
  a. The day before the flow experiment, aspirate the medium from the inlets.
  b. Add 120 μL EGM-2 containing 10 ng mL^-1^ TNFα to each channel inlets.
  c. Incubate for the required time and change medium to EGM-2.

***Note:*** For transmigration of human CTL and HUVEC 16–20 h of TNFα stimulation are optimal.

***Note:*** Other ECM proteins like collagen or cell attachment factors like L-poly-L-ornithine or poly-L-lysine can also be used. Several coating options are provided pre-coated by the vendor. Select appropriate coating (collagens I and IV, poly-L-lysine) or treatment (Ibitreat, hydrophilic) or untreated (hydrophobic) for your endothelial cells or experimental question before ordering. During culture time air bubbles can form in the channels. Prewarming media and equilibrating them in the same incubator before use in the μ channel limits the formation of air bubbles to a minimum. As cells do not grow at sites of air inclusion we remove them by slightly tilting the slide and tapping the side using a pipette stalk.

Troubleshooting 1, 2, and 3.

**CRITICAL:** HUVEC need to be prepared from frozen stock every week. Seeding cell numbers have to be determined empirically for your endothelial cells as proliferation rates can differ drastically even for the same cell type! Endothelial cells in continuous culture should never exceed 70% confluence! Ensure seeded cells distribute evenly by chaotically tilting the dish after seeding! Air bubbles in μ-channels can form after a while in the incubator. These can be removed by holding the μ-channel and gently tapping the long side using a pipette stalk repeatedly. It can take some time for the bubble to move initially, but it will work. Do not tilt the μ-channel too much when the lid is closed as the medium in the inlet ports can spill via contact with the lid. When tapping the μ-channel do not use excessive force, it can take some time for the bubble to move initially. Removing bubbles is essential as at locations of air bubble inclusion endothelial cells will not grow resulting in an inconsistent monolayer depending on the number of air bubbles. Bubbles mostly form during the day after seeding and should be removed latest the next day. When using other leukocytes than CTL for transmigration analysis optimal endothelial stimulation may differ. We found that HUVEC change their chemokine profile over the course of inflammation with CTL targeting chemokines being expressed highly after 16–20 h of TNF stimulation time (Schoppmeyer et al., 2022). This can differ for other endothelial cell types.

## Key resources table

**Table:** 

REAGENT or RESOURCE	SOURCE	IDENTIFIER
**Biological samples**		

Fresh blood, in heparin tubes, from voluntary healthy donors	Sanquin Blood Supply, Amsterdam, NL	N/A

**Chemicals, peptides, and recombinant proteins**		

Recombinant human tumor necrosis factor α, TNF-α	PeproTech	Cat#300-01A; Accession Number: P01375

**Experimental models: Cell lines**		

HUVEC, human umbilical vein endothelial cells, pooled	Lonza, Bornem, Belgium	Cat#C2519A

**Software and algorithms**		

Fiji/ImageJ, ImageJ v1.53	Schindelin et al., 2012	10.1038/nmeth.2019
Graphpad Prism 9.1.0 (221)	Graphpad Software, LLC	https://www.graphpad.com/scientific-software/prism/

**Other**		

Ficoll-Paque PLUS, density gradient media	GE Healthcare	Cat#17144003
CD8^+^ T cell isolation kit	Miltenyi Biotec	Cat#130-045-201
EGM-2 medium	Promocell	Cat#C-22011
EGM-2 supplement kit	Promocell	Cat#C-39216
RPMI1640, Glutamax	Thermo Fisher Scientific	Cat#61870036
Penicillin-Streptomycin (10,000 U/mL)	Thermo Fisher Scientific	Cat#15140122
Fetal bovine serum	Bodinco	N/A
Fibronectin solution	Sanquin Reagents	N/A
Trypsin-EDTA (0.5%), no phenol red	Thermo Fisher Scientific	Cat#15400054
μ slides VI (0.4)	Ibidi	Cat#80606
Human serum albumin, Albuman 200 g/L	Sanquin Reagents	N/A
Phosphate buffered saline (PBS)	Fresenius Kabi	Cat#8717973380153
Table-top pump (flow channel assays)	Prosense BV	Cat#NE-1010
Trypsin Neutralizer Solution	Thermo Fisher Scientific	Cat#R002100
Plastipak Syringe 60 mL Luer Lock	BD	Cat#309653

## Materials and equipment

### Flow buffer

**Table:** HEPES base buffer (in ddH_2_O)

20 mM	HEPES
132 mM	NaCl
6 mM	KCL
1 mM	MgSO_4_
1.2 mM	K_2_HPO_4_

Set to pH 7.4 with NaOH. This base buffer can be prepared in bulk and stored at 4°C.

**Table:** HEPES+ buffer

HEPES base buffer with addition of:	
1 mM	CaCl_2_ (from 1 M stock in H_2_O)
1 g L^-1^	D-glucose (weighted as powder)
0.5%	(w/v) human serum albumin (from 200 g L^-1^ stock)

After adding the additional reagents filter the buffer. Prepare fresh and use it within 1 day. Warm buffer to 37° in a water bath or incubator before performing the assay.

## Step-by-step method details

### Calculation of shear stress

Calculation of shear stress is described for the μ-channel VI 0.4 system. Formula for μ-channel VI 0.4 (differs for other channel types, see application note at Ibidi https://ibidi.com/img/cms/support/AN/AN11_Shear_stress.pdf): τ=η⋅176.1⋅Φ.

(Φ = flow rate mL/min; τ = shear stress dyn cm^-2^; η = dynamical viscosity of the flow medium dyn⋅s cm^-2^, see also Ibidi application note).

Dynamical viscosity (η) for μ-channel VI 0.4, $\eta \;=\;0.0072\;dyn\cdot s\;cm^{-2}$ (for aqueous solutions).

Solve for Φ: $\frac{\text{τ}}{\text{η∗}176.1}=\;\text{Φ}$. When 1 dyne cm^-2^ is to be applied to the channel set a flow rate of: $\frac{1{\text{ dyne cm}}^{-2}}{0.0072\text{ dyn}\cdot {\text{s cm}}^{-2}\text{∗ }176.1}=∼0.8{\text{ ml min}}^{-1}$.

You can now set a flow rate of 0.8 mL on the pump to achieve 1 dyne cm^-2^ of shear stress in the μ-channels. Physiological shear stress in venous cells ranges between 0.5 to 5 dyne cm^-2^ or more (Malek et al., 1999). We use 0.7 dyne cm^-2^ for CTL and other leukocytes as well.

***Note:*** The flow rate setting on the pump depends on the diameter of the used syringe. Ensure to set the correct diameter for syringe-based pump systems to apply the correct flow rate.

### Preparation of the flow system

**Timing: 1 h before start of the experiment**

The inflamed monolayer containing μ-channels are connected to the pump system and are flown with HEPES buffer for equilibration. Setting up the flow system is very straightforward, yet there are some points to note, especially the connection of the flow tubing to the channel inlets. Avoid inclusion of air bubbles at the inlet connected to the liquid container (opposite to the pump) as they will flow through the channel causing damage to the monolayer near instantaneously. This can be achieved by filling inlets to be connected until a meniscus forms at the top (1–2 mm high, Figure 2C). The flow system (filled with HEPES+ buffer) Luer connector can be filled up with buffer while holding it upside down to remove excess air. This greatly decreases occasions of air inclusion.

1. Place the μ-channel containing inflamed endothelial monolayers (check for consistent monolayer under cell culture microscope) to a microscope with phase contrast imaging and fix to a sample holder.
  a. Ensure the slide is fixed and remove the lid, cover all but the channel to be used with the lid by shifting it to the side (Figure 2B).
    ***Note:*** Avoids evaporation of medium from and contamination of the unused channels while imaging another.
  b. Connect the flow tubing (pump to channel and liquid container (HEPES+) to channel slide, includes clamps (Figure 2A, ∗) to block liquid flow when disconnecting tubes for attachment to channel inlets) via a small piece of tubing (Figure 2A, #).
2. Unlock the clamps and connect the pump to one side (Figure 2A, #) and the liquid container to the other (Figure 2A, Methods videos S6 and S7).
3. Flush the tubing (stored in 70% ethanol) with 50 mL water followed by 50 mL PBS and 50 mL HEPES+ (A high flow rate of 10 mL min^-1^ can be used as no cells are present) (Methods video S8).
4. Clamp the tubing (Methods video S9) on both sides and apply excess volume to the μ-channel inlet chambers of the channel to be imaged (Figure 2C).
5. Remove the connective tubing (Figure 2A, green #).
6. Connect the Luer connectors from the pump and container side to the channel (Figure 2D).
  a. Flush excess air out of the Luer connector using a 200 μL pipette tip and placing the opening facing up before connecting.
7. Flush the endothelial cells with HEPES+ buffer at ∼ 0.7 dyne cm^2^ for at least 10 min.

**CRITICAL:** Avoid air bubble inclusion during connection of the liquid container side Luer connector. Once connected removal of the Luer connectors will flush the channel with air bubbles and the channel cannot be recovered. Prepare excess channels in case this happens. Bubbles trapped at the pump side will flow away (unless the bubble is very large) towards the pump without passing the channel, thus can do no damage to the monolayer. For optimal flow conditions it is recommended to connect both Luer connectors without air inclusions. Fill Luer outlets with a 200 μL pipette tip and HEPES+ buffer to remove excess air before connecting. Also ensure during injection of CTL not to introduce air into the system as this can reach the monolayer and have destructive effects on the monolayer. Tap air out of the syringe carefully before injecting.

### Imaging transmigration under flow

**Timing: min to 1 h**

In this step image series of CTL flown over the inflamed monolayer of endothelial cells are recorded microscopically. For quantitative analysis 10× or 20× objectives are optimal using phase contrast (not DIC or brightfield) as the phase contrast change of transmigrated CTL can easily be identified. For imaging in higher detail many overlapping positions in the channel need to be recorded to record enough events with the lower field of view (63× or 100× objectives). At this higher magnification we recommend using fluorescent labels for CTL and HUVEC.

8. Prepare 1 mL of CTL at 1 × 10^6^ mL^-1^ in RPMI or HEPES+.
9. Use the microscope software position system and save several positions randomly on the channel connected to the flow system. Focus and use an auto-focus function if available.
  a. Avoid areas too close to the channel walls.
  b. Test the positions, lighting and focus settings by running the series for 2 or 3 rounds.
  c. Inject the CTL from the syringe slowly into the injection port (located within the liquid container side tubing, Figure 2A, ∗).

**CRITICAL:** Inject very slowly as fast injection will cause cells to flow back potentially into the liquid container,

***Note:*** Try to match the pump speed (for our pump and the used 60 mL syringes it holds (diameter 26.7 mm) a flow rate of 0.5 mL per min creates shear forces of around 1 dyne cm^-2^ which is close to the physiological flow rate in veins (Malek et al., 1999).

10. Start a live image and follow a position near the inlet from the liquid container.
11. Once CTL begin “raining” into the live frame (top to bottom or bottom to top depending on the pump orientation) start recording (15–30 min at 5–10 s intervals). Troubleshooting 1, 4, 5, and 6.

**CRITICAL:** Remaining focus is crucial for proper analysis especially at higher magnifications so ideally use an auto-focus solution. For CTL 15–25 min recording usually are enough for cells to transmigrate, this reduces the file size. Other cells like neutrophils and monocytes transmigrate even faster. We recommend 20 min as a sufficient time frame for CTL. If cell adhesion but no transmigration can be observed after 15 min, troubleshoot CTL activity and HUVEC inflammatory response.

### Image analysis and quantification of transmigration parameters

12. Open the saved image series in ImageJ. ImageJ can be freely downloaded from https://imagej.nih.gov/ij/download.html (Schindelin et al., 2012).
13. Open the counting tool and prepare and prepare an excel file.

**Figure 3:**
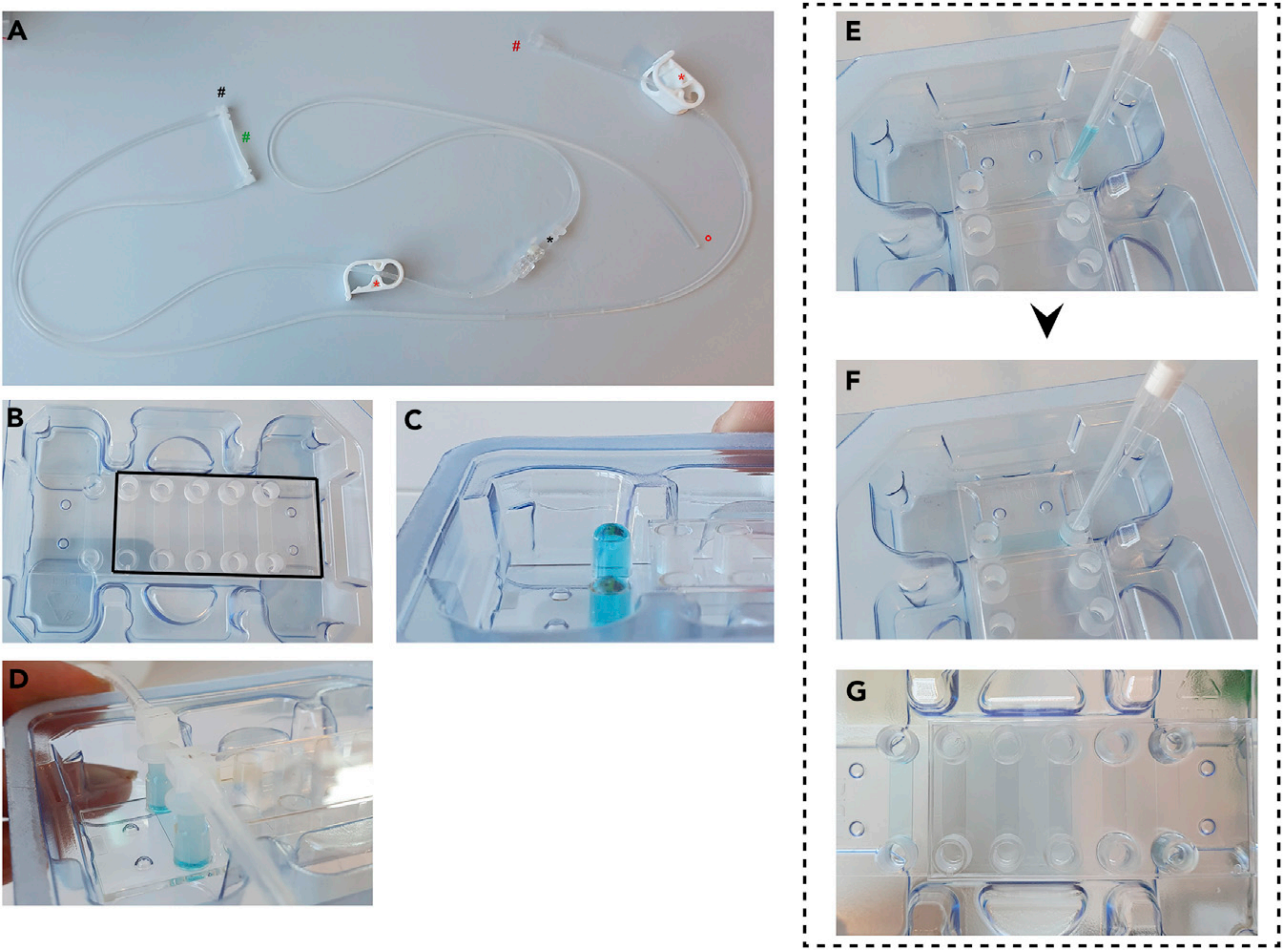
Flow channel setup (A) In (A) the complete tubing is shown as required for setting up the flow system. (B) Placement of the lid when using several channels for flow experiments. Black outline indicates shifted lid position. This avoids evaporation of the medium from the channels during recordings. The use of a humidifier in the microscope chamber is still recommended. (C) to properly attach the Luer locks from the flow tubing to the channel without trapping air bubbles in the inlets, apply medium to one inlet until a meniscus forms on both inlets (water was used for demonstrative purposes and colored blue for visibility). (D) Successful connected Luer connectors without air inclusion. (E–G) Coating solutions and cell suspension loading is performed by tilting the pipet tip towards the channel inlet at the bottom of the loading chamber (E). Add coating solution or cell suspension in one quick eject (F). Successful loading or seeding with homogenous distribution of the liquid and no air inclusions (G) (related to: Preparation of the flow system).

***Note:*** CTL adapt different morphologies when adhering to inflamed HUVEC, ranging from round (non-migratory) to spreading and protruding migratory phenotypes (Figure 3A).

***Note:*** Transmigrated CTL can be identified by a phase contrast change from bright to dark and consistent dark contrast when underneath the endothelium (Figure 3B).

14. Move the time slider to the end of the series and count the number of CTL in the image.
  a. This is the number of adherent cells.
  b. Use the ImageJ counting tool using different groups to count adherent, transmigrated and so on.
  c. Save the counts in an excel file.
15. Follow each cell individually CTL from adhesion through the timeline and visually identify transmigrated cells by change in phase contrast (Figure 3B).
  a. Mark the cells transmigration spot using the counting tool.
  b. This gives the number of all transmigration events (local and distant).

***Note:*** Ensure cells are transmigrated and not just flattened (CTL also turn dark but turn bright again later in their path).

***Note:*** Follow each transmigrated CTL individually to ensure it is properly transmigrated. This (flattening with increased phase contrast) is very rare in controls but can occur for certain experimental conditions, e.g., inhibiting adhesion factors (Schoppmeyer et al., 2022)) Example in Methods video S9 (see also Critical below).

16. Determine for each transmigrated cell how many EC:EC junctions they passed from their point of adhesion before transmigration (if they cross more than one junction, or equally pass several cell bodies, before transmigrating they are scored as distant diapedesis events).
  a. This gives the number of distantly transmigrated CTL.
    ***Note:*** This is important as we could show that transcellular diapedesis relies on the sequential local activation of adhering CTL via the diapedesis synapse (Schoppmeyer et al., 2022) and when synapse formation is inhibited CTL begin apical migration and show more distant events which are mostly paracellular.
  b. Local transmigration events: #ofalldiapedesisevents–#ofdistantlytransmigratedCTL=#localdiapedesisevents.
  c. Frequency of distant diapedesis events: #ofdistantdiapedesisevents/#ofalldiapedesisevents∗100=%ofdistantdiapedesisevents.

17. For local transmigration events determine the route of transmigration.
  ***Note:*** Transcellular migration is preceded by a rounded phenotype and does not include apical migration of the CTL after monolayer contact (Schoppmeyer et al., 2022).
  ***Note:*** Paracellular events occur at EC:EC junctions and are preceded by a polarized phenotype.
  a. These allow calculation of the percentage of each route of all local transmigration events.

18. Count the number of adherent rounded but not transmigrated nor apically migrating CTL (Figure 3C).
  a. Unpolarized fraction: #ofunpolarizedroundedcells/#ofadherentcells∗100=frequencyofunpolarizedcells.
    ***Note:*** High levels of unpolarized adherent cells indicate e.g., low CTL activity or quality, lack of chemotactic stimulation.
  b. Polarized fraction: #ofadherentcells−#oftransmigratedcells−#ofunpolarizedcells/#ofadherentcells=frequencyofpolarizednottransmigratedcells.
    ***Note:*** The number of apically migrating but not transmigrating CTL. Low transmigration and high polarization frequency point towards endothelial deficiency to provide transmigration signals e.g., actin dynamics, ICAM-1 clustering (see Methods video S10).

19. Calculate:
  a. Transmigration efficiency: numberoftransmigratedcells/numberofadherentcells∗100=X%.
  b. Frequency of distant diapedesis: numberofdistantlytransmigratedcells/numberoftransmigratedcells∗100=Y%.
  c. Transcellular route frequency: numberoftranscellularevents/numberoflocaltransmigrationevents∗100=Z%.
  d. Paracellular route frequency: 100%−Z%=A%.

***Note:*** ImageJ also provides automated and manual tracking plugins. TrackMate, ManualTracking, MTrackJ are examples of plugins which are part of the ImageJ/Fiji suite, but may require separate software to analyze the generated data. Depending on the plugin these may require fluorescent or binary images for segmentation and detection. Automated tracking in phase contrast images is not viable even with proprietary software (e.g., Imaris). We recommend these for tracking experiments to analyze migratory parameters like velocity and persistence using fluorescent labels. For scoring of behavior and morphology in phase contrast images manual analysis is still superior.

**CRITICAL:** It is important to follow cells over the whole time-course especially if they change contrast (dark contrast, sign of transmigration). Although this does not occur often in control conditions certain treatments can induce the following behavior (see Methods video S10 as example). In those cases, adhering CTL flatten and adapt a darker contrast but usually spread over a larger area than after actual transmigration. You can identify these flattened but not actually transmigrated cells by: 1. carefully observing the contrast as flat untransmigrated CTL show inhomogeneous contrast while transmigrated CTL show homogeneously dark contrast; and 2. Follow the CTL over the whole time course as when showing the described behavior CTL can change the contrast back to brighter values for a part of its migration path also changing its morphology to a migratory phenotype, even if briefly. Actually, transmigrated CTL will not resurface in this system despite its 2D limitations and tend to stay compact, without many protrusions or spreading, when underneath the endothelium!

## Expected outcomes

In our experience transmigration experiments with isolated human CTL show a transmigration efficiency of around 60% on average. About 50% of all local transmigration events are transcellular. The number of distant transmigration events is usually low around 5%–10% of all transmigrating CTL. Adhesion numbers (CTL adhering from flow per field of view) are largely dependent on effector and effector memory CTL frequency in the isolate but ranges between 10 to 30 for a 10 × objectives field of view at 1 × 10^6^ injected CTL. See Figures 1C–1F for example data from a control experiment. See Methods videos S1 and S2 for examples on inflamed (20 h) and uninflamed monolayers being flown with recovered CTL. Mophologies observed are the same under static conditions Methods video S11.

## Limitations

As an *in vitro* 2D assay limitations are posed by the plastic surface CTL encounter post diapedesis. The culture conditions, stimulation times and cell origin can drastically alter transmigration efficiency. 3D tissue matrix, subendothelial basement membrane and other cell types usually present in vasculature are missing. Cultured cells do not represent the full physiological context in vivo.

## Troubleshooting

### Problem 1

No T cells were visible after injection in the live image frame.

### Potential solution

If no cells are visible after a few minutes (depends also on the length of the tubing used) ensure the correct orientation of the tubing, that no air is trapped in the tubing and if the pump is set to withdraw not pump (which would flow the CTL away from the channel into the liquid container).

### Problem 2

Monolayer does not form after several days in culture in the μ-channel.

### Potential solution

This can have various reasons. Increase seeding density when seeding the channel. Make sure to change medium twice a day. Never aspirate the medium inside the channel while cells are growing inside! Ensure medium is fresh and contains all necessary supplements for your endothelial cells.

### Problem 3

Endothelial monolayer breaks open after inflammatory stimulation.

### Potential solution

This is a rare nuisance and potentially caused by non-confluent monolayers upon inflammatory stimulation. Consistently observe the cells during growth period with a cell culture microscope. Ideally they should reach confluence after 24 h followed by another 24 h of culture before inflammatory stimulation. This results in activated but consistent monolayers of endothelial cells.

### Problem 4

Air bubbles are formed in the tubing during experimental recording.

### Potential solution

Various factors apply. Degas solutions if possible before use. Ensure stable incubation conditions in the microscope chamber. Ensure the syringe is air free before injecting CTL. Tap the injection port during the initial flushing procedure as air can easily get trapped there and resist the general flow during preparation. Tap the whole tubing during initial preparation to loosen potentially stuck air bubbles.

### Problem 5

CTL do not adhere or transmigrate.

### Potential solution

Follow the isolation protocol precisely and stick to the recommended cell numbers and density. RPMI1640 can turn quickly and its pH becomes unstable. Use fresh RPMI and add 10 mM HEPES to the medium. FCS in the medium is essential for CTL to maintain proper function, 10% are sufficient. Check pH of the medium using a pH stripe and a few μL after recovery culture. If no adhesion occurs at all ensure TNFα is functional, check visually (HUVEC elongate after successful inflammatory stimulation, Figures 1A and 1B) and by checking ICAM-1 upregulation via flow cytometry or Western blot. The flow rate should not be too high, set flow rate to reach shear forces of 0.7–1 dyne cm^-2^. CTL numbers after recovery culture should not drop significantly (a 10% reduction compared to input is expected).

### Problem 6

CTL flow only at the side of the channel and show hardly any adhesion.

### Potential solution

This can be caused by a large air bubble blocking the inlet port on the container side. Injected CTL will be drawn to the sides of the channel and this results in no visible adhesion of CTL in the center areas. Ensure to connect the container side without trapped air bubbles.

## Resource availability

### Lead contact

Further information and requests for resources and reagents should be directed to and will be fulfilled by the lead contact, Rouven Schoppmeyer (r.schoppmeyer@gmail.com).

### Materials availability

No reagents or tools were generated for this analysis or experiments. All information about materials can be addressed to and will be addressed by the lead contact.

## Data Availability

No data was generated for this study. The experiments used were prepared in a different context. All data and information are available via the lead contact.
